# Editorial: EMG/EEG signals-based control of assistive and rehabilitation robots, volume II

**DOI:** 10.3389/fnbot.2023.1259773

**Published:** 2023-08-31

**Authors:** R. A. R. C. Gopura, Thilina Dulantha Lalitharatne, Dingguo Zhang

**Affiliations:** ^1^Department of Mechanical Engineering, University of Moratuwa, Moratuwa, Sri Lanka; ^2^Department of Medical Technology, University of Moratuwa, Moratuwa, Sri Lanka; ^3^School of Engineering and Materials Science, Queen Mary University of London, London, United Kingdom; ^4^Department of Electronic & Electrical Engineering, University of Bath, Bath, United Kingdom

**Keywords:** EMG signals, EEG signals, robot control, assistive robots, rehabilitation robot, robotic exoskeletons, sensors

Assistive and rehabilitation robots aid individuals with physical, cognitive, or sensory impairments, providing these individuals with opportunities for improved quality of life and independence. These robots make the life of a physically weak person more comfortable and are thus beneficial to society. Assistive robots possess exceptional abilities to recognize their surroundings, analyze sensory information, and carry out actions that are invaluable to individuals with disabilities. Furthermore, these robots play an important role in facilitating rehabilitation by understanding and enhancing the rehabilitation process. These robots aid a wide array of sensorimotor functions, provide therapeutic training, and evaluate the sensorimotor performance of patients with the highest precision.

In recent years, these systems have been developed, expanding the robotics field and its applications in a user-friendly manner. Furthermore, recent literature shows that many studies have been carried out to investigate the possibility of using biosignals to control assistive robots. Electromyography (EMG) and electroencephalography (EEG) signals of wearers have been extensively used to control assistive and rehabilitation robots since those signals can specifically be used to identify the motion intention of the wearer. Since EMG and EEG signals can represent the wearer's motion intention the wearer is free of actuating any additional device to control the assistive robot. However, the EMG and EEG derived from individuals with neurological impairments demonstrate different patterns when contrasted with those of their healthy counterparts.

Research on assistive robots integrates advanced mechatronics and intelligent sensing to restore weak sensorimotor functions. Assistive robots such as exoskeleton robots have been applied for tremor assessments and suppression and neurorehabilitation in recent studies. Furthermore, in the last few decades, many significant contributions have been made in this field. These robots still have a margin for development and advancement to enable a synergetic interaction with the user and to generate human-like motions.

The articles in the first volume of this Research Topic underlined both the promise and the challenges faced by the field of assistive and rehabilitation robots. The articles further recognized the need for additional perspective on the control of these robotic devices. Considering the significant responses received from the research community for the first volume of this Research Topic, the second volume also aims to create a multidisciplinary forum of discussion on the recent advances in controlling assistive and rehabilitation robots, such as exoskeleton robots. Authors have been invited to contribute high-quality articles containing original research findings, as well as systematic, narrative, or scoping reviews presenting relevant analysis and discussion on the current state of the art. Our main focus was topics that include but are not limited to developments of controllers for the upper limb/lower limb assistive/rehabilitation robots, biomechanical investigations of exoskeleton robots and other assistive/rehabilitation robots, novel sensors for assistive/rehabilitation robots to generate human-like motions, processing of EMG/EEG signals, EMG/EEG signals-based assistive/rehabilitation robot control, mathematical and physical algorithms for control of assistive/rehabilitation robots, assessment and benchmarking of assistive/rehabilitation robots' functionality, and clinical studies of assistive robot control.

The Research Topic received remarkable interest among researchers in the field. In this volume, a Research Topic of five meticulously selected articles that investigate various aspects of the subject are presented following a rigorous and thorough peer-review process. Among these articles, three articles discuss the utilization of EMG signals for the control of assistive and rehabilitation robots.

- *Exploring surface electromyography (EMG) as a feedback variable for the human-in-the-loop optimization of lower limb wearable robotics* by Grimmer et al.
- *A CW-CNN regression model-based real-time system for virtual hand control* by Qin et al.
- *LST-EMG-Net: long short-term transformer feature fusion network for sEMG gesture recognition* by Zhang et al.

Another article is dedicated to developing an EEG-sEMG hybrid strategy for prolonged active rehabilitation.

- *A low-cost and portable wrist exoskeleton using EEG-sEMG combined strategy for prolonged active rehabilitation* by Yang et al.

The article titled “*A robot-assisted therapy to increase muscle strength in hemiplegic gait rehabilitation* by Gil-Castillo et al.” presents the feasibility of using a robot-assisted therapy methodology based on the Bobath concept to perform exercises applied in conventional therapy for gait rehabilitation in stroke patients.

First, we discuss the contributions of articles that present the development of EMG-based control methods for assistive and rehabilitation robots.

- *Exploring surface electromyography (EMG) as a feedback variable for the human-in-the-loop optimization of lower limb wearable robotics* by Grimmer et al.

This research investigates whether lower limb sEMG could be an alternative feedback variable to overcome time-intensive metabolic cost-based exploration. Human-in-the-loop (HITL) optimization with metabolic cost feedback has been proposed to reduce walking effort with wearable robotics. An experiment was designed to elicit changes in the effort by loading and unloading pairs of weights in three randomized weight sessions for 13 subjects during treadmill walking. EMG of seven lower limb muscles was recorded for both limbs. Mean absolute values of each stride before and following weight loading and unloading were used to determine the detection rate for changing between loaded and unloaded conditions. The use of multiple consecutive strides and the combination of muscles to improve the detection rate were assessed and estimated the related acquisition times of diminishing returns. EMG drift was evaluated during the warm-up and the experiment to conclude on possible limitations of EMG for HITL optimization. The results suggest using EMG feedback of multiple involved muscles and from at least 10 consecutive strides to benefit from the increases in detection rate in HITL optimization.

- *A CW-CNN regression model-based real-time system for virtual hand control* by Qin et al.

Many strategies for motion classification or regression prediction tasks based on sEMG signals have been proposed. However, most of them are limited to offline analysis only. This article proposed a channel-wise-convolutional neural network (CW-CNN) regression model-based real-time control system for virtual hand control. An adaptive Kalman filter was designed to smooth the joint angles output before sending them as control commands to control a virtual hand. Three sessions of experiments were carried out with eight healthy subjects. The joint angles estimation accuracy and computational latency were analyzed during the real-time experiments. The experimental results show that the proposed control system has high precision for 3-DOF motion regression simultaneously, and the system remains stable and has low computational latency.

- *LST-EMG-Net: long short-term transformer feature fusion network for sEMG gesture recognition* by Zhang et al.

sEMG signal gesture recognition is widely used in rehabilitation therapy, human-computer interaction, and in many other fields. Deep learning has gradually become the mainstream technology for gesture recognition. It is essential to consider the characteristics of the surface EMG signal when constructing the deep learning model. However, current deep learning models usually extract features from single-length signal segments. This can easily cause a mismatch between the amount of information in the features and the information needed to recognize gestures. This is not conducive to improving the accuracy and stability of recognition. Therefore, in this research, a long short-term transformer feature fusion network (LST-EMG-Net) was developed. The network considers the differences in the timing lengths of EMG segments required for the recognition of different gestures. The researchers successfully fused the features using a feature cross-attention module and output the gesture category. The LST-EMG-Net was evaluated on multiple datasets based on sparse channels and high density.

Hemiparesis is a common consequence of stroke and has a serious impact on the quality of life of patients. Active training is an important factor in achieving optimal neurological recovery, but current wrist rehabilitation systems pose challenges in terms of portability, costs, and muscle fatigue risks during long-term use. The article on an EEG-sEMG hybrid control method presents developing an EEG-sEMG hybrid strategy for prolonged active rehabilitation. The article is titled “*A low-cost and portable wrist exoskeleton using EEG-sEMG combined strategy for prolonged active rehabilitation* by Yang et al.”. This article proposes a low-cost, portable wrist rehabilitation system with a control strategy that combines sEMG and EEG signals to encourage patients to engage in consecutive, spontaneous rehabilitation sessions. Furthermore, a detection method for muscle fatigue based on the Boruta algorithm and a post-processing layer is proposed. This method significantly improves the accuracy of fatigue detection for four distinct wrist motions.

- *A robot-assisted therapy to increase muscle strength in hemiplegic gait rehabilitation* by Gil-Castillo et al.

The above article examines the feasibility of using robot-assisted therapy based on the Bobath concept to perform exercises applied in conventional therapy for gait rehabilitation in stroke patients. The therapy aims to improve postural control and movement through exercises based on repetitive active-assisted joint mobilization, which is expected to produce strength changes in the lower limbs. Robotic assistance is gradually reduced as therapy progresses, and the patient's burden increases to achieve a certain degree of independence. The results showed a significant increase in hip abduction and knee flexion strength on both sides, although there was a general trend of increased strength in all joints. However, the range of motion at the hip and ankle joints was reduced.

This Research Topic provides a comprehensive overview of current research on the control of assistive and rehabilitation robots through EMG/EEG signals. The highlighted articles shed light on both the potential advancements and the limitations within the field of assistive and rehabilitation robotics. Furthermore, these articles recognize the need to provide further perspectives on the control methods of these robots. The researchers and practitioners should further concentrate on practical case studies, giving precedence to investigations and experiments that involve patients with neurological impairments. In this way, the quality of life for those requiring neuro-rehabilitation involvement can be enhanced.

Overall, the articles presented on this Research Topic not only contribute to clarifying the existing knowledge but also open the way to a new paradigm in the field of EMG/EEG signals-based control of assistive and rehabilitation robots. Through these articles, we aim to foster a deeper understanding of the role played by EMG/EEG signals in empowering assistive and rehabilitation robotic systems to improve human lives.

## Author contributions

RG: Writing—original draft, Writing—review and editing. TL: Writing—review and editing. DZ: Writing—review and editing.

